# Food and drink purchasing habits out of school at lunchtime: a national survey of secondary school pupils in Scotland

**DOI:** 10.1186/s12966-015-0259-4

**Published:** 2015-08-04

**Authors:** Jennie I. Macdiarmid, Wendy J. Wills, Lindsey F. Masson, Leone C A Craig, Catherine Bromley, Geraldine McNeill

**Affiliations:** Rowett Institute of Nutrition and Health, University of Aberdeen, Aberdeen, UK; Centre for Research in Primary and Community Care, University of Hertfordshire, Hertfordshire, UK; Institute for Health and Wellbeing Research, Robert Gordon University, Aberdeen, UK; Institute of Applied Health Sciences, University of Aberdeen, Aberdeen, UK; ScotCen for Social Research, Edinburgh, UK; Scottish Collaboration for Public Health Research and Policy, University of Edinburgh, Edinburgh, UK

**Keywords:** School, Children, Lunchtime, Food purchase, Supermarkets, Socio-economic deprivation

## Abstract

**Background:**

Food and drink purchasing habits of pupils out of school at lunchtime may be contributing to poor dietary intakes and overweight and obesity. The aim of this study was to identify the places from which purchases were made, types of food and drinks purchased and, the reasons for purchasing food or drinks out of school.

**Methods:**

A survey of the food and drinks purchasing habits of secondary school pupils (11-16 yrs) out of school at lunchtime was conducted in Scotland in 2010. A face-to-face interview and a self-completion questionnaire was designed to identify the food outlets used at lunchtime, types of food and drinks purchased and pupils’ reasons for purchasing food or drinks out of school. Height and weight were measured and BMI centiles used to classify pupils as normal weight, overweight or obese. Results were compared by age group, sex, BMI group and level of socio-economic deprivation.

**Results:**

Of the 612 pupils who completed the survey, 97 % reported having access to places selling food or drinks out of school at lunchtime, and of these 63 % made purchases. A higher proportion of pupils from more deprived areas reported purchasing food or drinks out of school, but the proportion making purchases did not differ significantly by sex or BMI group. Supermarkets were the outlets from which pupils reported most often making purchases, with fewer purchasing food or drinks from fast food takeaways, and this did not differ significantly by socio-economic deprivation. Reasons for making purchases included availability of preferred food and drinks, some of which are restricted for sale in schools, and social reasons, such as wanting to be with friends. Sandwiches and non-diet soft drinks were items most commonly purchased, followed by confectionery and diet soft drinks. However, less than 10 % of all the secondary school pupils reported purchasing these foods every day.

**Conclusions:**

Supermarkets, not just fast food outlets, should be considered when developing strategies to improve the dietary habits of pupils at lunchtime. The importance of food preferences and social reasons for purchasing food and drinks need to be acknowledged and integrated in future interventions.

## Background

Unhealthy eating habits are contributing to the high prevalence of overweight and obesity among young people in many countries. Thirty one percent of young people aged 12–15 years in Scotland [1] and 35 % of 12–19 year olds in the USA [2] are classified as overweight or obese. In the UK the intake of sugar (non-milk extrinsic sugars (NMES), similar to ‘free sugars’^1^) and saturated fatty acids among 11–18 year olds are substantially higher than the recommended population average [3, 4]. It is estimated that 30 % of NMES come from soft drinks, 20 % from table sugar, syrups, preserves (jams and honey) and confectionery (sugar and chocolate) and 10 % from fruit juices and smoothies [4]. The high consumption of soft drinks is not unique to the UK. In Scotland, 32 % of boys and 21 % of girls aged 15 years report drinking soft drinks daily, compared with 33 % of boys and 31 % of girls of the same age in the United States, and 45 % of boys and 31 % of girls in the Netherlands [5]. A reduction in consumption of soft drinks has been targeted in many public health strategies to improve dietary intakes, especially among children [6, 7].

Schools are considered an important place to provide an environment for healthy eating and this has become a focus for government policy in tackling poor eating habits and public health problems. All EU countries have some school food policies based on either mandatory regulations or voluntary guidelines to promote healthy eating, many of which include restricting the sale of sugar-sweetened beverages and sweet snacks [7]. In Scotland, the Schools (Health Promotion and Nutrition) Scotland Act 2007 set nutrient standards for school meals, prohibited the sale of specific food and drinks in schools (e.g. confectionery, sugar-sweetened beverages) and limits the sale of savoury snacks high in fat and salt [6]. After legislation or voluntary guidelines were set for schools the attention shifted towards the types of food and drinks available at outlets in the vicinity of schools. Since only 44 % of secondary school pupils in Scotland and 38 % in England eat lunch provided by their school [8, 9], it was viewed that the food and drinks purchased from other sources needs consideration.

It was estimated that there are an average of 23 ‘junk food outlets’ in the vicinity of every secondary school in England [10], with similar findings reported in Glasgow, Scotland [11]. Much of the research on purchasing food around schools has focused on the location and accessibility of takeaway and fast food outlets, particularly in deprived areas [12–14]. Concern about the proximity and easy access to fast food outlets being a contributory factor in the epidemic of obesity has stimulated much of the research, however the evidence to support this association is based mainly on observational studies [15–17].

Fast food outlets are only one of many places from which young people can purchase food or drinks out of school, and little is known about the usage of other outlets or the factors motivating purchases of food or drinks out of school. The aim of this research was to identify the range of food outlets that secondary school pupils (aged 11–16 years) access at lunchtime, to explore some of the factors associated with food and drink purchasing habits out of school at lunchtime, and to compare purchasing habits by age group, sex, BMI group and level of socio-economic deprivation.

## Methods

The Survey of Diet Among Children in Scotland (2010) assessed dietary intakes, prevalence of overweight and obesity and physical activity levels in a nationally representative sample of children and young people aged 3–16 years living in Scotland [18]. Children aged 8 years and older in the survey completed an additional module on food purchasing habits (FPH) out of school [19]. A detailed description of the survey methods has been published previously [18]. In summary, a sample of 3048 children were invited to take part in the survey, recruited using the Child Benefit records held by HM Revenue and Customs (national family welfare register), with data collected between June and November 2010. After an initial opt out period, the remaining 2712 participants were sent a food frequency questionnaire (FFQ) to complete, which was collected by a trained field worker when they visited the home to conduct a face-to-face interview. Interviews were completed with 1906 children and 1816 returned an FFQ. After exclusions for incomplete data (3 %) and extreme energy intakes (5 %) 1674 FFQs were included in the analysis. During the interview, socio-demographic characteristics were collected, the child’s height and weight measured and the FPH module administered. Six hundred and fifty six children were eligible to complete the FPH.

### Food purchasing habits

The FPH module for secondary school pupils comprised a combination of interviewer administered questions and a self-completion questionnaire [20]. A self-completion questionnaire was used because the interview was conducted in the presence of a parent or guardian and it was thought that some questions could be sensitive, for example some parents may not be aware their child was purchasing food or drinks out of school. The FPH module was designed for this survey and pre-tested using cognitive interviewing techniques to ensure usability and correct interpretation of the questions. It included questions about the pupil’s perceived opportunities to purchase food or drinks out of school, reported purchasing of food or drinks out of school, a description of the places food or drinks were purchased from and the reasons for choosing to purchase food or drink out of school. The majority of the questions were closed answer lists, which were developed using a combination of expert knowledge and feedback from the pilot interviews. Free text space was included for pupils to add an option not listed. When answering the questions pupils were asked to consider their purchasing habits in “a usual school week”. Purchases only included items bought by the pupil for themselves, and not items bought for them by other people.

### Dietary assessment

Habitual dietary intake was assessed using the Scottish Collaborative Group semi-quantitative FFQ (http://www.foodfrequency.org Accessed Jan 8 2015) which was completed by the pupils with help from their parent or guardian. They were asked to estimate the frequency of consumption of 148 food and drink items in a typical week, from one of nine options (ranging from ‘rarely or never’ to ‘seven or more times a day’). The relative validity of the FFQ for assessing NMES intake as percentage of food energy was assessed in a similar population, comprising a subsample of 153 children aged 3–17 yrs who completed both the FFQ and a 4-day food diary in an earlier survey [21]. There was no significant difference between median intakes of NMES (expressed as a percentage of food energy) from the FFQ and a 4 day food diary (16.0 % *vs.* 14.9 %).

### Weight status and socio-economic deprivation

Height and weight were measured and normal weight, overweight (not obese) and obesity were defined as a BMI <85th_,_ ≥85th to <95th and ≥95th percentiles, respectively, based on the 1990 UK centile charts [22]. Socio-economic deprivation was assessed using the Scottish Index of Multiple Deprivation (SIMD), which provides a relative measure of deprivation by area based on home postcode. It combines indicators over seven domains including income, employment, health, education, skills and training, housing, geographic access and crime [23]. SIMD is presented as quintiles of deprivation, with SIMD quintile 1 being the highest level of deprivation.

### Data analysis

Statistical analysis was carried out using the complex surveys module in SPSS Statistics version 19.0 (SPSS Inc., IBM Company, USA). The data were weighted to take account of selection and non-response bias [18], but unweighted sample numbers are presented in the tables. Six hundred and twelve pupils returned the FPM from which four pupils were excluded as they reported they did not have access to places that sell food and drinks out of school, and therefore lacked the opportunity to purchase food or drinks at lunchtime. The variation in sample size for the different analyses is explained by missing data for individual questions, or excluded data for questions that were completed incorrectly, e.g. more than one option selected when respondents were asked to select only one option. Differences in the proportion of pupils who reported purchasing food or drinks out of school by age group, sex, quintiles of SIMD and BMI group were assessed using the Pearson chi-squared statistic for complex samples. Habitual dietary intakes of energy, total fat, saturated fatty acids and NMES, expressed as a percentage of food energy, were compared between pupils who reported purchasing food or drinks out of school at lunchtime and those who did not using general linear models adjusted for age, sex and SIMD quintile.

### Ethics approval

The protocol for the study was reviewed and given favourable opinion by the National Centre for Social Research Ethics Committee (Application: P7070 Scottish Children’s Diet Survey 2010). Informed consent was obtained from both the parent/guardian and the child prior to participating in the study.

## Results

Six hundred and fifty six secondary school pupils aged 11–16 years were eligible to participated in the FPH survey, of whom 612 (93.3 %) completed it, with the majority (n = 608, 96.5 %) reporting that there were places close to their school from which they could purchase food or drinks at lunchtime. Table 1 shows the percentage of pupils with access to such places who ‘ever’ bought food or drinks out of school, which was derived from the question “do you ever buy food or drink out the school grounds at lunchtime?” The frequency of purchasing could therefore vary between pupils, for example from daily to only very occasionally Sixty-three percent of pupils with access to places selling food or drinks reported that they purchased food or drinks out of school at lunchtime. Pupils living in more deprived areas and older pupils (aged 13–16 years) were more likely to report purchasing food or drinks out of school at lunchtime, but purchasing did not differ significantly by BMI group or sex.

**Table 1:** Percentage of pupils (with access) who report *ever* purchasing food or drinks out of school at lunchtime

	n	%	p-value
All*	608	63.0	
Sex			0.077
boys	304	66.1	
girls	304	59.1	
Deprivation (SIMD)			0.015
1 (most deprived)	104	76.0	
2	122	64.0	
3	139	60.5	
4	113	63.0	
5 (least deprived)	130	52.6	
BMI groups**			0.106
not overweight or obese	392	62.5	
overweight, not obese	89	70.0	
obese	103	55.3	
School year			0.002
1 (11-12 yrs)	148	63.2	
2 (12-13 yrs)	145	71.5	
3 (13-14 yrs)	99	70.5	
4 (14-15 yrs)	103	52.8	
5 & 6 (15-16 yrs)	113	49.2	

*sample of 608 excludes 4 pupils who reported that they did not have access to places selling food or drinks out of school at lunchtime. **BMI groupings: not overweight or obese <85th percentile, overweight, not obese ≥85th to <95th percentile, obese ≥95th percentile

Some pupils purchased food or drinks from several different places and collectively the most common places were supermarkets, newsagents and sandwich shops/bakeries. Fewer pupils made purchases at takeaway and other fast food outlets. When asked which one place they most often made purchases, supermarkets were the most commonly reported, with a quarter of pupils reporting making purchases there, followed by sandwich shops/bakeries (Table 2). The places from which pupils reported purchasing food or drinks did not differ significantly by socio-economic deprivation. In addition to purchasing food or drinks at lunchtime, 73.4 % of pupils reported being able to access places selling food or drinks out of school at break times, and of those with access 23.8 % reported purchasing food or drink at this time. Boys rather than girls (31.9 % *vs* 15.4 %, p < 0.001) and older pupils (S1 to S5: 18.7 %, 14.0 %, 25.2 %., 24.8 %, 33.3 % p = 0.042) were more likely to purchase food or drinks out of school during break times but this did not differ significantly across deprivation groups.

**Table 2:** Food outlets from which pupils report *most often* or *ever* purchasing food or drinks at lunchtime

	*Most often* purchase	Outlets pupils *ever* purchase food or drinks**						
	% pupils (n = 262)	% pupils (n = 374)	Deprivation (SIMD quintile)					
			Least deprived (n = 76)	4 (n = 88)	3 (n = 60)	2 (n = 65)	Most deprived (n = 85)	p-value
Supermarkets	25.4	41.1	42.7	50.1	43.6	33.8	35.6	0.285
Sandwich shop or bakery	19.3	42.4	48.7	49.6	38.6	42.6	33.4	0.250
Takeaways, fast food, chip shops	17.2	36.5	33.8	29.0	31.1	46.6	41.2	0.133
Newsagents, sweet shops	12.2	49.0	42.6	46.3	37.7	53.1	60.2	0.083
Grocery or corner shops	8.6	27.8	25.5	21.3	30.4	37.3	27.0	0.271
Cafe, coffee shops, restaurants	5.9	15.0	16.5	9.2	22.0	18.1	12.6	0.278
Burger, chip or ice cream van	4.3	16.3	10.9	11.6	14.1	16.8	25.7	0.066
Garage, petrol station	2.2	12.0	9.8	10.3	19.6	19.2	5.5	0.055
Other*	5.0	9.1	10.0	5.1	11.2	10.7	9.6	0.739

*Includes sport centre, healthy food van, pharmacy, post office, **pupils could report more than one place

### Reasons for purchasing food or drinks out of school and for leaving the school

Reasons for purchasing food or drinks out of school and for leaving the school grounds at lunchtime are shown in Table 3. The most common reasons given for purchasing food or drinks from the places where they most often made purchases related to liking the taste and variety of foods sold in these outlets. Value for money and the proximity of the outlet to the school were other important determining factors. Fifteen percent of pupils reported that they bought food at these outlets because they thought they were healthy. The most common reasons given for leaving the school grounds at lunchtime to purchase food or drinks were to be with friends and to get food that was unavailable in school. Only a small minority of pupils thought that school meals were expensive and gave this as a reason for purchasing food or drinks out of school.

**Table 3:** Reasons why pupils purchase food or drinks out of school at lunchtime

Reasons pupils purchase food or drinks out of school from the places they *most often* purchase food or drinks	% pupils (n = 367)	Reasons pupils leave the school to purchase food or drinks	% pupils (n = 369)
There is a wide choice of food	53.6	Because my friends do	44.2
I like the taste of the food they sell	49.9	Because I can’t get the food I want in school	39.5
It is close to the school	45.0	The canteen queue is too long	38.3
It is good value for money	43.5	I want to get out of school	36.2
I can get food there that I can’t buy at school	37.9	I don’t like school lunches	35.4
My friends buy from the same place	32.5	I don’t like the canteen	26.7
I get some exercise	32.5	I like to choose where I spend my money	25.7
I get served quickly	29.8	It’s my right to choose where I go and buy my food	24.3
I like the look of food they sell	23.1	The food I want in the canteen runs out too quickly	20.2
The food is healthy	15.3	Canteen food is too expensive	12.3
My parent/guardian tells me where to go	0.6	I want to get a break from other people	5.6
Other reasons	2.1	I get treated with respect at shops out of school	4.1
		So adults can’t supervise what I buy	2.0
		Other reasons	6.7

### Types of food and drinks purchased out of school

Sandwiches and non-diet soft drinks were the items most commonly purchased daily (Table 4). Non-diet soft drinks were reported to be purchased every day by 12.6 % of pupils and at least once a week by 60.5 % of the pupils who reported ever purchasing food or drink out of school at lunchtime. Confectionery was reported as purchased everyday by 7.6 % of pupils and at least once a week by 68.6 % of pupils reporting to ever purchase food or drinks. To put the prevalence of purchasing by secondary school pupils into a wider context, of those who completed the FPH module and had the opportunity to purchase food and drinks out of school (n = 608), only 7.8 % and 4.7 % reported buying non-diet soft drinks or confectionery, respectively, out of school every day at lunchtime. However 91 % and 98 % of pupils of this age in the survey reported consuming these items, respectively, which were also the largest contributor to NMES in the diet of pupils in survey (16 % and 13 % respectively) [18].

**Table 4:** Percentage of pupils purchasing different types of food or drinks out of school at lunchtime

	Pupils who ever purchase out of school (%)				All pupils (%)
	(n = 367)				(n = 600)
	5 times per week	3-4 times per week	1-2 times per week	Rarely or never	5 times per week
Foods					
sandwiches	10.0	13.8	34.3	41.9	6.3
sweets or chocolate	7.6	15.2	45.7	31.4	4.7
pizza, chips or burger	4.8	13.6	39.8	42.3	5.0
crisps	5.3	8.8	32.4	53.5	3.3
fruit	4.3	5.2	23.2	67.2	2.7
cereal bars or biscuits	0.7	6.1	22.8	70.4	0.4
ice-cream or lollies	0.5	2.7	7.8	88.9	0.3
Drinks					
non-diet soft drinks	12.6	17.2	30.6	39.5	7.8
diet soft drinks	9.0	13.7	28.3	48.9	5.6
plain water	7.4	13.3	29.4	50.0	4.6
plain or flavoured milk	3.5	6.4	11.9	78.3	2.2
fruit juices or smoothies	2.9	7.6	19.6	69.9	1.8

### Dietary intakes

The intake of NMES (as percentage of food energy) in the habitual diets of pupils who ever purchased food or drinks out of school at lunchtime was higher than those pupils who did not make purchases out of school (mean (95 % CI): ever 18.6 % (17.7-19.4) *vs*. never 16.6 % (15.7-17.5) p = 0.011). Total energy intake (ever 7.82 MJ (7.51-8.13) vs never 7.11 MJ (6.73-7.49) p = 0.050) was slightly higher for those who purchased food or drinks out of school but total fat (as a percentage of food energy) (ever 33.1 % (32.5-33.6) vs never 32.4 % (31.8-33.0) p = 0.219)) and saturated fatty acids (as a percentage of food energy) (ever 13.2 % (12.9-13.5) vs never 12.9 % (12.6-13.3) p = 0.318)) did not differ significantly between pupils who reported purchasing food or drinks out of school at lunchtime and those who did not.

## Discussion

This study found that most secondary school-aged pupils in Scotland have access to places selling food and drinks out of school at lunchtime, with approximately two thirds of them making purchases at some time. Older pupils and those from more deprived areas were more likely to purchase food or drinks out of school at lunchtime; nevertheless more than half of all pupils in every sub-group reported purchasing food or drinks out of school at lunchtime at some time point. Supermarkets, followed by sandwich shops and bakeries, were the places at which pupils reported most often making purchases at lunchtime, irrespective of level of deprivation. The main reasons given for purchasing food or drinks out of school related to the variety of foods available and liking the foods that were sold, some of which are banned for sale in school. However, reasons for going out of school at lunchtime related more to social issues, for example to be with friends and wanting to leave the school grounds. The food and drinks sold in school were generally not considered expensive, but purchasing food out of school was seen by many as representing value for money.

Previous research shows that pupils with greater access to food outlets around schools were more likely to purchase food and drinks out of schools [14], however the present study found that almost all secondary school pupils across Scotland have access to places selling food or drinks at lunchtime and break times. Interestingly, in this study, supermarkets, rather than takeaway and fast food outlets, were the places from which pupils reported most often purchasing food or drinks at lunchtime. Previous research has focused on fast food outlets in the vicinity of secondary schools [13, 24], but in this study they were not the only or the main places where pupils were buying food or drinks at lunchtime. A UK study of changing retail environments around schools suggests that whilst overall numbers of food outlets have not changed in recent years, there has been an increase in the number of grocery stores (including supermarkets) within 800 metres of schools [25]. Supermarkets tend to provide a wider range of foods than many takeaway outlets, which may represent both healthy and less healthy choices. However, the importance of considering the wider food environment and not focusing just on takeaways and fast food outlets was highlighted in a recent study, when the association between exposure to takeaways and body weight disappeared when adjusted for supermarket exposure [26]. The role of supermarkets in influencing the diet should not be overlooked even among young people; a recent study showed that 65-75 % of added sugars consumed in the US are bought in supermarkets or grocery stores for example [27]. It has been reported that in supermarkets in the UK have more shelf space allocated to the sale of energy dense snacks (e.g. crisps, confectionery) compared with eight other high income countries and have the second highest ratio of space for snack foods to fruit in supermarkets (1.31:1) [28]. In the current study, however, we were not able to identify which foods or drinks were being purchased at the different types of food outlets, but this could be explored in future research.

The types of food or drinks pupils are buying out of school at lunchtime, e.g. soft drinks and confectionery, are some of the items prohibited for sale in schools by legislation. The restriction on the sale of these food and drinks may explain why some pupils buy these items out of school [29]. Sugary items such as these have long held attraction for children and young people and it will be difficult to restrict the consumption of them [30]. Leaving the school grounds at lunchtime was also related to social and environmental factors; pupils reported wanting to be with friends and not wanting to stay in school, for example. This is consistent with previous research that shows that the physical and social environment within schools (e.g. crowded and unpleasant cafeteria environments) are reasons that pupils leave to buy food or drinks out of school [31–33]. There is a strong social and cultural value to spending time with peers, including selecting what food or drink to buy when together and then consuming these purchases whilst walking back to school; rejecting such opportunities may be viewed as socially risky by young people [34, 35]. Food has an important symbolic and social meaning among teenagers, which is often more important than the nutritional properties and can make foods either more appealing or distasteful [36]. Refusing to purchase the food and drink available in school and electing to pursue other options is also an indicator of a young person’s growing autonomy and of them becoming an active consumer in the local marketplace [37]. Our findings show that older pupils are more likely to purchase items at local food outlets than younger pupils, who are often still adjusting and growing into the idea of autonomy at lunchtime [31, 38]. Such behaviour may be reinforced when it resonates with practices or values experienced within the family setting [39, 40]. These wider socio-environmental issues need to be acknowledged and incorporated into the development of future policies and interventions if pupils are to be encouraged to stay in school and to eat the food and drink provided there, when there are strong social reasons for them not doing so.

Despite concerns about obesity and the contribution of food purchased out of school, similar to the findings of Héroux *et al*. [24], this study did not show an association between BMI group and purchasing food or drinks out of school at lunchtime. There were no differences in habitual intakes of energy, total fat or saturated fatty acids between those who did and did not make purchases out of school at lunchtime. Although the habitual intake of NMES was higher among pupils who purchased foods or drinks out of school at lunchtime than those who did not, it was not possible to determine if this was a consequence of purchases made at lunchtime or different habitual eating patterns. Despite this, the mean intake in both groups exceeded the recommended population average for NMES suggesting that future public health strategies and interventions need to extend beyond the focus on schools and lunchtime purchases. However, the contribution is relatively small with less than 10 % of all pupils surveyed purchasing confectionery or non-diet soft drinks at lunchtime every day.

As with all surveys, the data are based on self-reported information from participants and in this survey pupils’ reports of food and drink purchases out of school at lunchtime may be under- or over-reported. Causal links cannot be inferred and we cannot assume that all purchases were consumed. Care was taken when designing the module to minimise any effect of pupils not wanting to disclose their purchasing habits in front of their parents by using a self-completion questionnaire. These data are from a nationally representative sample of secondary school-aged pupils in Scotland and provide an important observation of food and drink purchasing from food outlets within the vicinity of schools at lunchtime.

## Conclusions

Consideration of the places from which pupils purchase food and drinks out of school at lunchtime needs to go beyond the focus on fast food outlets since pupils in this survey reported most often purchasing food or drinks at supermarkets. The percentage of secondary school pupils who purchase high sugar foods every day, such as non-diet soft drinks and confectionery, out of school at lunchtime was less than 10 %. This questions the emphasis, effort and likely impact of changing the food environment around schools on improving the overall diet of young people and tackling obesity. It remains important to improve purchasing habits at lunchtime at school but wider public health strategies are needed to improve the dietary intakes of young people across the whole day. While schools can play an important role in educating young people about healthy eating, across the year school days account for only one in three of all lunches eaten and approximately one in eight of all meals, assuming three meals a day are eaten. Future interventions and policies to improve the diets of young people also need to acknowledge and integrate the importance of the social aspects and peer pressures associated with food and eating practices both within and beyond the school gate.

## Endnote

^1^Non-milk extrinsic sugars are ‘free sugars’ (all sugars added in manufacturing, cooking, or at the table, and those in honey, syrups, fruit juices and fruit purees) plus 50 % of the fruit sugars in dried, canned and stewed fruit.
